# Joint EANM/SNMMI/ESTRO practice recommendations for the use of 2-[^18^F]FDG PET/CT external beam radiation treatment planning in lung cancer V1.0

**DOI:** 10.1007/s00259-021-05624-5

**Published:** 2022-01-13

**Authors:** Sofia C. Vaz, Judit A. Adam, Roberto C. Delgado Bolton, Pierre Vera, Wouter van Elmpt, Ken Herrmann, Rodney J. Hicks, Yolande Lievens, Andrea Santos, Heiko Schöder, Bernard Dubray, Dimitris Visvikis, Esther G. C. Troost, Lioe-Fee de Geus-Oei

**Affiliations:** 1 Nuclear Medicine Radiopharmacology, Champalimaud Centre for the Unkown, Champalimaud Foundation, Lisbon, Portugal; 2 Department of Radiology, Leiden University Medical Center, Leiden, The Netherlands; 3 Department of Radiology and Nuclear Medicine, Amsterdam University Medical Center, Amsterdam, The Netherlands; 4 Department of Diagnostic Imaging (Radiology) and Nuclear Medicine, University Hospital San Pedro and Centre for Biomedical Research of La Rioja (CIBIR), Logroño (La Rioja), Spain; 5 Henri Becquerel Cancer Center, QuantIF-LITIS EA 4108, Université de Rouen, Rouen, France; 6 Department of Radiation Oncology (MAASTRO), GROW – School for Oncology, Maastricht University Medical Centre, Maastricht, The Netherlands; 7 Department of Nuclear Medicine, University of Duisburg-Essen and German Cancer Consortium (DKTK)-University Hospital Essen, Essen, Germany; 8 The Sir Peter MacCallum Department of Oncology, University of Melbourne, Melbourne, Australia; 9 Radiation Oncology Department, Ghent University Hospital and Ghent University, Ghent, Belgium; 10 Nuclear Medicine Department, CUF Descobertas Hospital, Lisbon, Portugal; 11 Molecular Imaging and Therapy Service, Memorial Sloan Kettering Cancer Center, New York, USA; 12 Department of Radiotherapy and Medical Physics, Centre Henri Becquerel, Rouen, France; 13 QuantIF-LITIS EA4108, University of Rouen, Rouen, France; 14 LaTIM, INSERM, UMR1101 Brest, France; 15 Department of Radiotherapy and Radiation Oncology, Faculty of Medicine and University Hospital Carl Gustav Carus, Technische Universität Dresden, Dresden, Germany; 16 OncoRay – National Center for Radiation Research in Oncology, Faculty of Medicine and University Hospital Carl Gustav Carus, Technische Universität Dresden, Helmholtz-Zentrum Dresden-Rossendorf, Dresden, Germany; 17 Helmholtz-Zentrum Dresden - Rossendorf, Institute of Radiooncology - OncoRay, Dresden, Germany; 18 National Center for Tumor Diseases (NCT), Partner Site Dresden, Germany: German Cancer Research Center (DKFZ), Heidelberg, Germany; Faculty of Medicine and University Hospital Carl Gustav Carus, Technische Universität Dresden, Dresden, Germany; Helmholtz Association / Helmholtz-Zentrum Dresden – Rossendorf (HZDR), Dresden, Germany; 19 German Cancer Consortium (DKTK), Partner Site Dresden, and German Cancer Research Center (DKFZ), Heidelberg, Germany

**Keywords:** Radiotherapy, EANM, SNMMI, ESTRO, 2-[^18^F]FDG PET, CT, Radiation therapy, Planning, Lung cancer

## Abstract

**Purpose:**

2-[^18^F]FDG PET/CT is of utmost importance for radiation treatment (RT) planning and response monitoring in lung cancer patients, in both non-small and small cell lung cancer (NSCLC and SCLC). This topic has been addressed in guidelines composed by experts within the field of radiation oncology. However, up to present, there is no procedural guideline on this subject, with involvement of the nuclear medicine societies.

**Methods:**

A literature review was performed, followed by a discussion between a multidisciplinary team of experts in the different fields involved in the RT planning of lung cancer, in order to guide clinical management. The project was led by experts of the two nuclear medicine societies (EANM and SNMMI) and radiation oncology (ESTRO).

**Results and conclusion:**

This guideline results from a joint and dynamic collaboration between the relevant disciplines for this topic. It provides a worldwide, state of the art, and multidisciplinary guide to 2-[^18^F]FDG PET/CT RT planning in NSCLC and SCLC. These practical recommendations describe applicable updates for existing clinical practices, highlight potential flaws, and provide solutions to overcome these as well. Finally, the recent developments considered for future application are also reviewed.

## Introduction

### Radiotherapy in lung cancer

#### Lung cancer

Lung cancer is a major cause of cancer death in both men and women, with an incidence of 11.6% and mortality of 18.4% worldwide (World Health Organization cancer report 2020 [1]). Despite declining in incidence, it is estimated to remain the leading cause of cancer deaths in the USA in 2040 [2]. The two main types of lung cancer are non-small cell lung cancer (NSCLC) and small cell lung cancer (SCLC).

NSCLC represents more than 80% of lung cancer cases and includes two subtypes: (a) non-squamous (including 40% adenocarcinoma, 5–10% large-cell carcinoma, and other subtypes) and (b) 30% squamous cell (epidermoid) carcinoma [3]. Since 2017, NSCLC is staged according to the eighth edition of the IASLC (International Association for the Study of Lung Cancer) in tumour, nodes, and metastases (TNM) based on the American Joint Committee on Cancer (AJCC) staging system [4]. Patients are grouped in stages I, II, III, and IV. Approximately 55% of cases have distant metastases at diagnosis, while roughly 30% present with locally advanced disease, including mediastinal lymph node involvement [5].

SCLC represents fewer than 20% of lung cancers. Despite the fact that TNM staging [6] also has been proposed for SCLC, it is commonly classified in two clinical stages based on the possibilities of including the disease in radiotherapy (RT) fields: (a) limited stage that typically includes TNM stage I to III and (b) extensive stage that includes TNM stage IV (presence of metastases), but also cT3-4 tumour (multiple lung nodules) and/or tumour/nodal volume that is too extended to be encompassed in a tolerable radiation plan. Around 66% of SCLC cases present with metastatic disease [7, 8].

#### Radiotherapy

External beam radiation therapy (EBRT) focuses radiation, mainly high energetic photons, but sometimes electrons, protons, and heavy ions, from outside the body onto the tumour. Newer EBRT techniques enable lowering the radiation dose to nearby healthy tissues. These include the following: (a) Intensity modulated radiation therapy (IMRT) and volumetric modulated arc therapy (VMAT) are an advanced form of three-dimensional conformal radiation therapy. Using inverse treatment planning, beams from different angles are shaped according to the target form and their intensity is adjusted throughout the treatment to optimize dose to the target while limiting dose to surrounding normal tissues.

(b) Stereotactic body radiation therapy (SBRT), also known as stereotactic ablative RT (SABR), is most often used to treat the primary tumour only, particularly in early-stage lung cancer and increasingly used to treat oligometastatic disease. Instead of giving a small dose of radiation (typically 2 Gy) each day for several weeks (usually 4-5), SBRT uses focused beams of high-dose radiation (typically 6–18 Gy) in fewer (usually 2–8) treatment sessions. Such plans achieve a high biological effectiveness, i.e., introduce a high level of tumour cell kill while sparing the surrounding tissues.

#### Common clinical indications for RT in lung cancer

The following recommendations for RT in lung cancer are based on the National Comprehensive Cancer Network (NCCN), the European Society of Medical Oncology (ESMO), and the Advisory Committee for Radiation Oncology Practice of the European Society for Radiotherapy and Oncology (ESTRO-ACROP) guidelines [5, 8–12]. In NSCLC, RT is recommended in the following situations:

- Early-stage disease — SBRT as primary treatment in stage I and selected node-negative stage IIA disease when patients are medically inoperable or when patients refuse surgery. In case of positive pathological margins, postoperative RT is also advocated.
- Locally advanced NSCLC — depending on the age and comorbidity of the patient, concurrent or sequential chemoradiotherapy (CRT), or RT alone, is the standard in inoperable (node-positive) stage II disease and in unresectable stage III disease. Yet, even in potentially resectable cases, decisions on the optimal local treatment strategy — either surgery or RT — will be based on expected benefits and side effects. RT will be delivered with three-dimensional conformal radiotherapy or more commonly with IMRT.
- Advanced/metastatic NSCLC — local palliation or prevention of symptoms (such as pain, bleeding, or obstruction of vessels or bronchi) or definitive local therapy to unifocal or oligo-metastases (the latter most frequently being addressed with SBRT);

Furthermore, RT also has a role in the two stages of SCLC, as part of either definitive or palliative therapy, as follows:

- Limited stage SCLC — concurrent CRT, ideally delivered with twice daily RT sessions, is the treatment of choice for stage IIB–III SCLC, although a sequential approach may be preferred in case of an initial volume that is not amenable to RT or in a patient unfit to tolerate such an intensive treatment scheme. In rare cases, when resection revealed unexpected positive lymph nodes in patients with clinical stage I–IIA SCLC, postoperative loco-regional RT will be considered.
- Extensive stage/metastatic SCLC — consolidation thoracic RT after partial or complete response to systemic therapy, especially if there is low burden of extrathoracic metastatic disease.

Both in limited stages responding to chemotherapy and in extensive disease stages without progression after chemotherapy, prophylactic cranial irradiation has been shown to be of benefit and should be offered to the patient.

#### Selective versus elective nodal irradiation

According to ESTRO-ACROP guidelines [8, 12], elective nodal irradiation is not recommended. Selective nodal irradiation (e.g. lymph nodes with proven metastatic involvement or highly suspicious on imaging) instead of elective nodal irradiation (e.g. all lymph node territories included in the primary tumour drainage) gives the opportunity of increasing the dose to the involved lymph nodes, while reducing toxicity [13–15].

Studies have found a low incidence of isolated nodal recurrence after selective nodal irradiation, in both NSCLC [16–18] and SCLC [19, 20], even in the era of highly conformal treatment techniques, demonstrating the usefulness and safety of this approach. In NSCLC, selective nodal irradiation on the basis of CT or 2-[18F]fluoro-2-deoxy-D-glucose (2-[^18^F]FDG) positron emission tomography (PET) resulted in isolated nodal failures in fewer than 5% of cases [16]. In SCLC, 2-[^18^F]FDG PET-based selective nodal irradiation achieved a lower rate of isolated nodal failures (3%) compared to CT (11%) [20].

### The role of PET/CT in lung cancer diagnosis and treatment

#### PET/CT imaging

A PET/CT system is an integrated imaging device, capable of acquiring both PET and CT scans. Reconstructed PET and CT images are spatially co-registered with the caveat that the CT is acquired very rapidly, while the PET is usually acquired in multiple steps over several minutes. PET/CT fusion is the simultaneous display of co-registered CT and PET images. The CT component of a PET/CT scan can be acquired with variable parameters (e.g., mAs, kVp, pitch, with or without contrast) to suit the clinical need or according to local protocols and regulations, for instance using a low-dose, low-resolution CT scan only for attenuation correction and anatomical localization, or a higher-dose, higher-resolution CT if greater anatomic detail is required.

2-[^18^F]FDG PET/CT is a standard imaging modality for staging, selection for curative RT, defining and delineating the target volume in the RT planning phase, and detection of residual or recurrent disease. 2-[^18^F]FDG PET/CT can also be used for treatment response assessment, and it is the strongest and independent predictor of overall survival after RT [21–25].

#### 2-[^18^F]FDG PET/CT for lung cancer staging

2-[^18^F]FDG PET/CT is widely used in lung cancer staging, because 2-[^18^F]FDG is avidly taken up by the primary tumour, lymph nodes, and distant metastases. In a prospective multicentre trial, 2-[^18^F]FDG PET/CT changed management strategies in approximately 72% of cases [26]. Since 2-[^18^F]FDG PET/CT has higher staging accuracy than CT alone, it may reduce healthcare costs by avoiding unnecessary RT or surgery, enabling better selection of patients amenable to curative treatment intent and reducing toxicity [21, 27].

Regarding NSCLC, integrated 2-[^18^F]FDG PET/CT provides the best non-invasive means for staging and is more accurate and cost-effective than non-PET/CT approaches [21, 28]. The sensitivity and specificity to detect mediastinal lymph node involvement on CT are reported as 50–70% and 65–85%, respectively, whereas the corresponding values on 2-[^18^F]FDG PET/CT are 75–85% and 85–90% [29, 30]. In a study of 167 patients with apparent stage I-III NSCLC by conventional imaging, PET detected metastases with increasing frequency from stage I (7.5%) through stage II (18%) to stage III (24%, *p* = 0.016) [31]. A prospective multicentre randomized trial verified that combining preoperative 2-[^18^F]FDG PET/CT with a conventional workup prevented unnecessary thoracic surgery in 20% of patients [30].

In SCLC, 2-[^18^F]FDG PET/CT also increases staging accuracy, and it is superior to CT alone [32–36]. A systematic review and meta-analysis published in 2019 (including 6 studies and 277 patients) concluded that 2-[^18^F]FDG PET/CT was superior to conventional staging, with a pooled percentage of staging changes (either from limited-stage to extensive-stage disease, or vice versa) in 15% of patients [36]. In another study, 2-[^18^F]FDG PET/CT upstaged 19% of patients and downstaged 8% of patients [37].

The limitations of 2-[^18^F]FDG PET/CT include (a) suboptimal brain staging due to high 2-[^18^F]FDG uptake in normal cerebral tissue. Magnetic resonance imaging (MRI) continues to be the primary modality to detect brain metastases; (b) uptake in reactive or granulomatous nodes and in infectious processes, which may usually be recognized by experienced readers based on the distribution of abnormality (pattern recognition) or CT images; (c) subcentimeter nodules, mucinous adenocarcinomas with a relatively small amount of cells, and low-grade malignancies are insufficiently detected; (d) chest wall invasion assessment is suboptimal; and (e) respiratory blurring causing misregistration between the PET and CT components, particularly at the lung bases, which may be addressed by respiratory motion correction techniques (see “Respiratory motion correction”).

According to the ESMO guidelines about NSCLC [9], correct diagnostic work-up is necessary to detect regional lymph node metastases prior to multidisciplinary management.

When abnormal mediastinal and/or hilar lymph nodes are found on CT and/or PET, endoscopic (endobronchial) ultrasound [E(B)US] is recommended over surgical staging. EUS-guided fine needle aspiration complements 2-[^18^F]FDG PET by improving the overall specificity and positive predictive value to 100%, with an overall accuracy of 97% [38]. Based on data from 5 meta-analyses, Peeters et al. [39] calculated that the addition of E(B)US can decrease the false negative rate of 2-[^18^F]FDG PET/CT (from 13 to 3% in enlarged nodes, and from 6 to 1% in normal-sized nodes). However, for 2-[^18^F]FDG PET/CT-positive but E(B)US-negative nodes, the false negative rate of E(B)US was as high as 14–16%. Therefore, these authors recommended to include such PET+/EBUS− nodes in the RT planning volume. Moreover, since a negative EBUS cannot rule out metastatic disease reliably, they suggested proceeding to surgical staging/mediastinoscopy if PET findings are highly suspicious for mediastinal invasion.

The joint guideline by the European Society of Gastrointestinal Endoscopy (ESGE), together with the European Respiratory Society (ERS) and the European Society of Thoracic Surgeons (ESTS) [40], for the diagnosis and staging of lung cancer, recommends that EBUS be performed in peripheral NSCLC without clear mediastinal involvement on CT or PET/CT if at least one of the following apply: (i) enlarged or 2-[^18^F]FDG-avid ipsilateral hilar nodes [since proven nodal involvement may change the target volume], (ii) primary tumour without 2-[^18^F]FDG uptake [since 2-[^18^F]FDG is thus not reliable for staging], or (iii) tumour size ≥ 3 cm (a priori higher risk for metastatic nodal disease).

The ESMO guidelines on SCLC [10] also recommend excluding mediastinal lymph node involvement if a surgical approach is an option for patients with limited stage.

#### 2-[^18^F]FDG PET/CT for lung cancer RT planning

##### Target volumes in RT

**Figure. 1:**
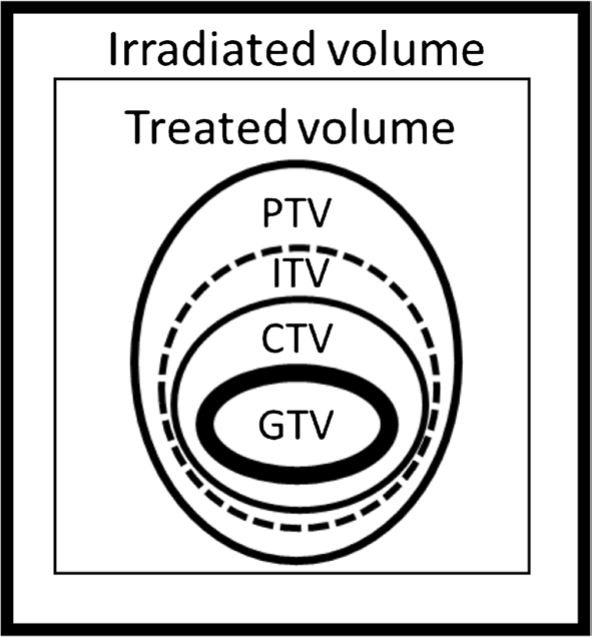
GTV, CTV, PTV, and ITV schematic definitions (based on the International Commission on Radiation Units and Measurements, report 62 - ICRU-62)

In RT planning, it is important to define the target lesion and to delineate the following volumes [41]:

(a) Gross tumour volume (GTV) includes the lesion that can be imaged.

(b) Clinical target volume (CTV) contains the GTV plus a margin for subclinical disease spread, which cannot be imaged.

(c) Internal target volume (ITV) is the margin needed around the CTV to compensate for possible motion or deformation of the CTV, considering respiratory motion.

(d) Planning target volume (PTV) ensures that the RT dose is delivered to the CTV, compensating for systematic and random uncertainties during treatment planning or delivery. (Fig. 1)

##### 2-[^18^F]FDG PET/CT for target volume definition and delineation

2-[^18^F]FDG PET/CT plays an important role in RT planning of lung cancer [42–44]. It improves tumour definition and has the advantage of reducing inter- and intra-observer variation when used to guide target volume delineation [45, 46]. Target volume definition entails the identification of all recognizable tumour locations, allowing the delineation of the GTV of the primary tumour and GTV of the lymph nodes separately, if anatomically distinguishable. During image interpretation, the challenge is to define those tumour and/or nodal volumes that should be included in the GTV, thus aiding in their subsequently delineation and discrimination from organs at risk (OAR). Usually, metabolic information from PET is used to identify tissues that contain tumour, and the anatomic information from CT is used to delineate (the margins of) the primary tumour and lymph nodes provided that there is sufficient contrast to define these margins [44].

RT planning using 2-[^18^F]FDG PET/CT is particularly helpful in identifying tumour boundaries in case of extra-thoracic or mediastinal tumour extension, when the tumour and normal tissue have similar visual appearance on CT, and when there is atelectasis caused by compression of the airways by tumour (enabling the discrimination between collapsed lung and tumour) [44, 47]. In lesions with high 2-[^18^F]FDG uptake intensity, the spill over effect can artificially increase apparent GTV beyond that confirmed by anatomical boundaries. This situation can be solved using different contrast, level, and window settings on both PET and CT imaging [48, 49]. The pre-set lung window setting (approximate window — *W* = 1600 and level — *L* = -600) should be used to delineate tumour surrounded by lung tissue, while the mediastinum pre-set window setting (approximate *W* = 400 and *L* = 20) should be used to delineate lymph nodes and primary tumour invading the mediastinum or chest wall [12]. Although there are no validated quantitative approaches for PET contouring, the procedure can be improved with visual calibration of the W/L settings, for example, standardizing signal intensity visually according to the normal background, or using linear grayscale for PET images alone. For PET/CT image fusion, it is recommended to use a linear scale with one or at most two colours [44].

The time between staging 2-[^18^F]FDG PET/CT and start of RT should not exceed 3 weeks, because disease may progress rapidly, invalidating prior target definition [8, 12, 44].

In patients having undergone neoadjuvant/induction chemotherapy prior to radiation treatment planning, 2-[^18^F]FDG PET/CT scan prior to chemotherapy needs to be taken into account when identifying metastatic lymph nodes. Lymph nodes fulfilling the above mentioned criteria for inclusion in the target volume (see “2-[^18^F]FDG PET/CT for lung cancer staging”), still need to be included irrespective of their 2-[^18^F]FDG uptake or appearance on CT after chemotherapy. For inclusion in the target volume, the initial 2-[^18^F]FDG PET/CT is to be registered in the subsequent 2-[^18^F]FDG PET/CT acquired in radiation treatment position. Caution must be taken regarding geometrical alignment as well as CT dose calibration.

The recent multicentre, randomized, controlled PET-PLAN Trial (ARO-2009-09, NCT00697333) confirmed the safety of using 2-[^18^F]FDG PET/CT to define the target for primary tumour and selective nodal treatment in patients with locally advanced NSCLC undergoing CRT [15]. The study showed that the mean total RT dose was significantly higher in the 2-[^18^F]FDG PET-based target group than in the conventional target group, allowing doses of 68 Gy or more to be achieved more frequently (47% vs. 33% of cases). The risk of loco-regional progression in the 2-[^18^F]FDG PET-based target group was lower than in the conventional target group (14% vs. 29%), without increasing toxicity.

Considering the lack of information in the literature specifically about SCLC, the following paragraphs about primary tumour and lymph node definition and delineation will mainly focus on NSCLC.

###### Primary tumour

2-[^18^F]FDG PET/CT has the advantage of increasing inter- and intra-observer reproducibility. This enables the reduction of the primary tumour GTV in at least 13–17% of patients compared with CT-measured tumour volume [50, 51].

The Phase II prospective trial by the Radiation Therapy Oncology Group, RTOG 0515 [52] demonstrated that 2-[^18^F]FDG-derived tumour volumes were significantly smaller than those derived by CT alone (86.2 vs. 98.7 mL), resulting in RT planning modification.

A systematic review and meta-analysis [53] estimated that the use of 2-[^18^F]FDG PET/CT imaging for RT planning purposes led to changes in target definition in 36% of cases (43% in NSCLC and 26% in SCLC) and a change of treatment intent from curative to palliative treatment in 20% of cases (22% in NSCLC and 9% in SCLC).

###### Lymph nodes

Several reasons for false negatives and false positive lymph nodes on 2-[^18^F]FDG PET imaging are widely reported in literature [54]. For instance, tumours with low cellular density (such as carcinoid, mucinous, and lepidic adenocarcinoma histology) or subcentimeter in size may not show 2-[^18^F]FDG uptake higher than background. Conversely, areas of inflammation or infection, including granulomatosis (e.g. tuberculosis, sarcoidosis, and Langerhans cell histiocytosis), pneumoconiosis (e.g. asbestosis, anthracosis, and silicosis), and post-surgery and post-irradiation fibrosis may show 2-[^18^F]FDG uptake unrelated to tumour.

There is consensus that 2-[^18^F]FDG PET be used to define lymph nodes included in the GTV. Although pathological confirmation was not systematically obtained in every study, several authors concluded nodal staging by 2-[^18^F]FDG PET improved GTV definition and delineation, enabling dose intensification to involved nodes, while reducing irradiation and resultant toxicity to normal tissues. The RTOG 0515 trial [52] reported that 2-[^18^F]FDG changed nodal GTV contours in 51% of patients.

A few studies compared the target definition based on CT and/or 2-[^18^F]FDG to surgical information, which was considered the gold standard. Vanuytsel et al. [55] observed that the inclusion of 2-[^18^F]FDG PET/CT information changed nodal GTV in 62% of patients compared to CT information, and improved GTV coverage compared to pathological data from 75% with CT alone to 89% with 2-[^18^F]FDG PET/CT. Nevertheless, true nodal GTV may still be underestimated, in particular in higher TNM stages [56].

The delineation of regional nodal disease on PET has been conducted in similar ways as that for the primary tumour. Taking all available information into account, it is recommended that 2-[^18^F]FDG positive lymph nodes only be omitted from the RT plan in the setting of a representative negative nodal biopsy, for instance, showing granulomatous disease [39, 57]. Nestle et al. [58] reported improved inter-observer agreement for mediastinal involvement on 2-[^18^F]FDG PET/CT after a standardized training process for PET readers.

##### 2-[^18^F]FDG PET/CT for response evaluation and residual or recurrent disease detection

Local tumour recurrence after RT usually occurs within 2 years after treatment and represents a diagnostic challenge. According to the NCCN, ESMO, and ESTRO-ACROP guidelines [5, 9, 11], follow-up imaging after lung cancer surgery or SABR should be done with chest CT. The selective use of 2-[^18^F]FDG PET is recommended when recurrence is suspected based on serial CT scans, to differentiate true malignancy from benign conditions, such as atelectasis, consolidations, and radiation-induced fibrosis.

2-[^18^F]FDG PET/CT may help to differentiate recurrent tumour from post-radiation fibrosis if sufficient time has elapsed since last treatment, to avoid false positive uptake due to inflammation [59, 60]. Considering the low positive predictive value of 2-[^18^F]FDG PET, pattern recognition is important for detecting recurrence. The areas treated with SBRT can have well-defined intense 2-[^18^F]FDG uptake up to 6 months after treatment, and low-level, ill-defined uptake can last up to 2 years because of radiation pneumonitis in the surrounding lung parenchyma [61, 62]. Moreover, local recurrence tends to be more focal, whereas inflammation has a more diffuse appearance [63, 64]. 2-[^18^F]FDG uptake and structural lung parenchyma changes in geographic distribution concordant to the prior radiation treatment area may assist in differentiating between these entities. Software that allows fusion of radiation dose-volume contours as DICOM-object with PET images can be particularly helpful in this regard. Moreover, the 2-[^18^F] FDG uptake pattern suggesting post-radiation pneumonitis may precede symptoms [65, 66], while oesophageal toxicity causing clinical symptoms can be detected as increased linear 2-[^18^F]FDG uptake along the oesophagus [65, 67].

The reduction in 2-[^18^F]FDG accumulation at the tumour site after treatment indicates tumour response and is associated with better prognosis [68–70]. A decrease in 2-[^18^F]FDG uptake may be an earlier indicator of response to treatment, occurring before a decrease in tumour size. The greater the decline in uptake, the better the response. Considering that 2-[^18^F]FDG PET/CT has a high negative predictive value, a residual uptake equal to or below background is defined as a complete metabolic response [68, 70]. However, it is debatable whether background is better evaluated in liver or blood pool. Metabolic response criteria, such as the PET Response Evaluation Criteria in Solid Tumours (PERCIST), that assess change in standardized uptake value (SUV) corrected for lean body mass (SUL) have also been used to quantify metabolic response to treatment in clinical trials [71]. The Hopkins criteria consider focal 2-[^18^F]FDG uptake greater than that of the liver (scores of 4 and 5) to represent residual disease [72]. Considering possible false-positive findings on 2-[^18^F]FDG PET, patients suitable for salvage therapy should undergo a biopsy for confirmation because it remains difficult to differentiate fibrosis from residual disease or local recurrence. Currently, there is not a validated method or reference cut-off SUVmax number to accurately differentiate responders from non-responders.

##### 2-[^18^F]FDG PET/CT for predicting outcome after RT

Some studies have suggested that the presence of heterogeneous tumour uptake on baseline 2-[^18^F]FDG PET/CT can predict local failure and can therefore be used to define areas at risk of recurrence after treatment [73, 74].

Usmanij et al. [24] verified that changes in metabolic parameters could predict response to concomitant CRT as early as the end of the second week of treatment in patients with locally advanced NSCLC; i.e. a total lesion glycolysis (TLG) decrease ≥38% was associated with a significantly longer one-year progression free survival (80% vs. 36%). In a meta-analysis, Na et al. [23] reported that the SUVmax in the primary tumour both before and after RT was able to predict patient outcome with regard to local control and overall survival. Other studies have also shown that post-treatment response assessment with 2-[^18^F]FDG PET/CT can predict survival [24, 75, 76].

## Goal

The aim of this guideline is to provide general information about 2-[^18^F]FDG PET/CT in lung cancer (both NSCLC and SCLC) and specific considerations for RT planning with an emphasis on the collaboration between nuclear medicine physicians and radiation oncologists. In this guideline, concepts about target definition, target delineation, and per-treatment evaluation will be included.

This field is rapidly evolving, and this guideline may rather be appreciated as a dynamic document than a definitive document, nor is it a summary of all existing protocols. Local variations should be taken into consideration when applying this guideline, preferably in a multidisciplinary setting.

## Qualifications and responsibilities of personnel

### Physicians

RT planning for lung cancer is at the intersection of radiation oncology, nuclear medicine, and diagnostic radiology expertise. It has been shown that mutual training and close collaboration of specialists from these fields optimize the RT target delineation process [77]. It is important to consistently check hybrid images co-registration before any target delineation and a joint GTV delineation by a radiation oncologist together with a nuclear medicine physician and radiologist, produced encouragingly consistent results [78].

Treatment planning includes professionals trained in multimodality imaging according to interdisciplinary training programs, who are also participating in the lung multidisciplinary tumour board. Specific training programs, including case-based training (e.g. ESTRO target volume determination courses, https://www.estro.org/Courses), should be consulted to facilitate exchanges between specialties.

The nuclear medicine physician or PET/CT specialist confirms that the radiopharmaceutical administration and image acquisition are according to the guidelines [42] and verifies that the acquired image is adequate for medical diagnosis. Informed consent might need to be obtained for 2-[^18^F]FDG PET/CT, according to national/institutional requirements.

The target volume definition and delineation for treatment planning is performed by the radiation oncologist. Where radiation oncology departments own a PET/CT scanner and conduct their own simulation scans, it is recommended that staff performing the target delineation are properly trained in 2-[^18^F]FDG PET/CT image interpretation. In any case, consultation of a radiologist and/or nuclear medicine physician should be easily accessible, for example when in doubt about possible physiologic uptake or abnormal findings during the delineation process.

Different approaches are proposed in the interpretation and GTV delineation (see “Interpretation and target volume delineation”). Therefore, it is recommended to develop departmental instructions for GTV delineation, which should include testing of the reproducibility of metabolic GTV delineation within a nuclear medicine department. All delineation steps for GTV should be performed or supervised by radiation oncologists according to local practice. An additional peer review by another radiation oncologist is highly recommended because inter-observer variation in the delineation is one of the main uncertainties in RT planning of lung cancer.

### Technologists

PET/CT scans should be performed by a qualified registered and/or certified nuclear medicine technologist [79]. If specific PET knowledge and training have been gained by nuclear medicine technologists, they should be able to perform PET quality control testing. It is advisable that technologists receive and maintain training in each other’s fields to create a group of professionals with complete competence to acquire PET/CT scans in the RT setting. In practice, cooperation between departments and personnel is fundamental to guarantee adequate execution of the protocol.

Imaging technologists are responsible for proper patient preparation to achieve optimal 2-[^18^F]FDG biodistribution, tracer administration according to radiation safety requirements, adequate handling of radioactive patients during the imaging procedure, appropriate PET/CT image acquisition and reconstruction, and acquisition of the planning CT scans (with or without intravenous contrast). The technologists trained in RT planning are also involved in the imaging process. They are responsible for installing the RT equipment on the PET/CT (e.g. flat bed, treatment positioning devices) and for ensuring stable and reproducible positioning of the patient confirming that it is suitable for RT planning according to the region of the body to be treated and guarantying patient’s comfort. This also includes respiratory gating, if available and validated at the site, and marking of the isocentre reference points on the patient. The patient positioning is of utmost importance to avoid mistakes and pitfalls that interfere with the RT planning and treatment, some common human situations that should be bared in mind are the site of treatment, isocentre position, inserted references and measurements, time of contrast and bolus administration, and any additional medication. A multidisciplinary approach of both nuclear medicine and RT technologists is needed when verifying image quality and applicability for RT planning.

### Physicists and information technology personnel

A multidisciplinary and collaborative approach should also apply to physicists, information technology personnel, and technical support team. Quality control of the PET/CT should be done by a medical physicist with special expertise in nuclear medicine. PET/CT scanners must adhere to regional, national, and international quality standards, including international dosimetry and radiation precautions for patients and staff alike. A further task is to develop and implement more refined and reproducible methods of PET/CT segmentation (automatic algorithms and/or artificial intelligence-based) to improve the detection of lung lesions. A medical physicist should ensure adherence to good practice, perform radiation dose monitoring, and develop algorithms to minimize the radiation exposure [80, 81]. Quality control of the RT equipment should be done by a physicist with expertise in RT. The physical RT planning and the dose calculation should be reviewed by a dedicated physicist before the final treatment plan has been approved.

## Procedure and specifications of the examination

As the availability of imaging modalities assisting RT planning is variable between institutions and continuously evolving, embedding 2-[^18^F]FDG PET/CT imaging in the RT plan is considered in the ESTRO-ACROP current guidelines [8, 11, 12], but should be tailored to local workflow. The workflow should be defined and managed in a multidisciplinary manner. Several specific aspects need to be considered in 2-[^18^F]FDG PET/CT-based treatment planning of lung cancer. These will be described in the following paragraphs.

### Request

The acquisition and interpretation of imaging studies are guided by the clinical questions that need to be answered. The request for a 2-[^18^F]FDG PET/CT in RT position should be written (preferably digitally) and contain all standard information for an oncological 2-[^18^F]FDG PET/CT, in particular, location of the (former) primary lung tumour and/or known metastases, previous radiation therapy dates, dose and locations, and previous/simultaneous chemotherapy regimens prescribed. Information about other lung diseases (e.g. tuberculosis, sarcoidosis, and other granulomatous diseases, pneumonia), prior talc pleurodesis, thoracic surgery, or recent biopsy should also be provided. It should explicitly include the request for performing the scan in the RT position. In most cases, the administration of intra-venous contrast will be requested, and, in these cases, kidney function (or glomerular filtration rate) and history of contrast allergy should be noted.

### Patient preparation and precautions

Patient preparation should be done according to the “2-[^18^F]FDG PET/CT EANM procedural guidelines for tumour imaging version 2.0” and the American “Society of Nuclear Medicine and Molecular Imaging (SNMMI) procedure guideline for tumour imaging with 18F-FDG PET/CT 1.0” [42, 82]. This includes fasting for at least 4 h prior to imaging, proper hydration, verification of a serum glucose level <11 mmol/L, and resting in a quiet and warm environment during the 2-[^18^F]FDG-uptake time that should ideally last 60 ± 5 min.

The administration of intravenous contrast improves primary tumour delineation, regional lymph nodes identification, and OAR definition on CT. In such cases, kidney function and history of contrast allergy should be verified before intravenous contrast injection. The administration of contrast media and premedication should follow local chest CT radiological protocols.

If no diagnostic thoracic CT is available, it should be considered to include an additional low-dose, deep-inspiration thoracic CT to adequately evaluate the lung parenchyma. This is also important for comparison with previous or later thoracic CTs.

Patient setup should be performed with levelling lateral and sagittal lasers, to ensure accurate alignment and positioning. Reference ink or tattoo marks of the isocentre should be used (one on the right side, left side, and ventral centre) to ensure reproducibility of setup at the time of treatment [83].

### Radiopharmaceuticals

The administration of 2-[^18^F]FDG should follow the EANM/SNMMI guidelines about PET/CT imaging in the oncology context and should be in concordance with the “As Low As Reasonable Achievable (ALARA)” principle, which may enable the reduction of administered activity, mainly in the newest generation scanners [42, 82, 84].

### Hardware

According to the International Atomic Energy Agency (IAEA) consensus report 2014, the PET/CT scanner should be equipped with a flat RT table top, patient positioning devices, and the CT component should be calibrated for its safe use in RT planning and dose calculation [44, 85]. The EORTC recommendations for RT planning in lung cancer state that a stable and reproducible patient positioning is essential [86]. If possible, patients should be in supine position with both arms above the head. It is recommended to use support devices for arms and knees to improve the position reproducibility and patient comfort. The equipment used for patient immobilization should be similar when performing PET/CT and RT. The PET/CT imaging should be verified before contouring to avoid co-registration misalignment, even in hybrid PET/CT systems.

### Protocol/image acquisition

2-[^18^F]FDG PET/CT performed from the mid-thighs to skull base, after bladder voiding, is recommended, and the acquisition details should follow the EANM/SNMMI procedural guidelines and also the specifications of the PET/CT scanner used [42, 82]. When 2-[^18^F]FDG PET/CT is performed for staging with the possibility of doing RT planning in one setting, a flat RT table-top should be used. Patients should be informed about the need to place tattoo marks.

### Respiratory motion correction

Respiratory motion may have impact on tumour localization, delineation, SUV quantification, and, consequently, dose delivery in RT. Despite being highly dependent on the implementation of the PET manufacturer, based on the “Report of the American Association of Physicists in Medicine Task Group 76” [87], respiratory motion correction may be organized in the following four categories:

1. Motion-encompassing methods include (a) slow CT scanning, (b) four-dimensional (4-D) CT/respiration-correlated CT, and (c) forced shallow breathing with abdominal compression.
  In slow CT acquisition, multiple respiration phases are averaged per slice. The disadvantage is the increased dose compared with conventional CT scanning and the loss of resolution due to motion blurring, and therefore, it is not recommended for lung tumours that are adjacent to either the mediastinum or the chest wall.
  A suitable solution for obtaining high-quality CT data in the presence of respiratory motion is 4-D CT or respiration-correlated CT. The 4-D CT could be combined with a 3-D or a 4-D PET scan. In 4-D acquisitions, the scans are retrospectively binned into a number of breathing cycle phases, using a respiratory tracking system [44, 88]. The impact of additional 4-D PET information to 3-D PET is promising but is still a matter of active investigation [89–92], with several translational research projects within prospective SBRT trials, such as the Freiburg mono centre phase II STRIPE trial or the EORTC 2113-0813 Lungtech trial [93, 94]. A limitation of 4-D CT is that it may be affected by variations in respiratory patterns during acquisition and various techniques are currently being investigated to reduce these respiratory artefacts [95, 96].
  The forced shallow breathing with abdominal compression technique employs a stereotactic body frame with an attached plate or an inflatable belt that is pressed against the abdomen. The applied pressure to the abdomen reduces diaphragmatic excursions, while allowing limited normal respiration. Implementation of these techniques is highly dependent on patient cooperation, and previous training can help increasing image quality.
2. Controlled breathing methods include (a) moderate or deep-inspiration breath-hold, (b) active-breathing control, (c) self-held breath-hold without respiratory monitoring, and (d) self-held breath-hold with respiratory monitoring [97, 98].
  Moderate or deep-inspiration breath-hold is advantageous because it significantly reduces respiratory tumour motion and changes internal anatomy (the diaphragm pulls the heart posteriorly and inferiorly) in a way that often protects critical normal tissues.
  Breathing control is a method that enables reproducible breath-hold. After a nose clip is put on, the patient breathes through a mouthpiece connected via flexible tubing to a spirometer according to the technologist’s instructions. The patient breathes normally through a device consisting of a digital spirometer to measure the respiratory trace. In active breathing control, the patient is connected to a balloon valve.
  Self-held breath-hold with or without respiratory monitoring means that patients hold their breath at some point in the breathing cycle according to a respiratory monitor device or voluntarily, respectively.
3. Respiratory gating includes gating based on (a) external respiration signal or (b) internal fiducial markers that are implanted in or close to the tumour using percutaneous or bronchoscopic techniques. It involves image acquisition in a defined part of the breathing cycle, and the gating characteristics are established according to the patient’s respiratory motion [99].
  The gating PET/CT imaging improves the assessment of intra-tumour heterogeneity and may be adequate for dose painting [100] (see “Imaging tumour metabolism and dose painting”).
4. Data-driven gating techniques — instead of using hardware-driven motion correction strategies (as described in previous sections), new methods are being explored using data-driven software analysis. Some examples include (a) motion characterization directly from a patient’s gated scan using the signal to create a single optimal bin, and leading to conformal adaptive imaging [101], and (b) motion information extraction from the reconstructed images [102]. The real-time data-driven motion correction, as opposed to post-processing methods, represents an important innovation in the speed of processing data for clinical practice [103].
  Currently, there is no robust evidence to choose one method over the other, so the decision is based on the availability in the department and its implementation should follow institutional and national regulations.

### Interpretation and target volume delineation

2-[^18^F]FDG PET/CT images should be discussed between the nuclear medicine physician, the radiologist, and the radiation oncologist to define the best treatment planning. Several PET-based tumour volume delineation methods have been evaluated, and algorithms for semiautomatic 2-[^18^F]FDG PET segmentation have evolved exponentially in the last decade [104, 105]. Since 2017, artificial intelligence-based segmentation approaches seem to outperform previously state-of-the-art algorithms [105, 106]. Classifications of PET auto-segmentation methods can be based on image processing algorithms, pre- and post-processing steps, and automation level [105]. Some of the possible segmentation methods are summarized in Table 1, and may include the following four groups:

1. Manual methods — visual interpretation and manual delineation of PET-based GTV using a computer mouse slice-by-slice are common in daily practice and widely used [107]. One of the main disadvantages in manual delineation of PET images is its strong operator dependence that can, therefore, result in high intra-observer variability and reduced reproducibility [108].
2. Threshold-based segmentation methods — they are commonly used because of their simplicity. They rely on a fixed or adaptive threshold (e.g. SUV, background noise, contrast, signal-to-noise ratio, size), above which all voxels are considered to belong to the tumour volume. Fixed thresholding alone should be avoided since it strongly depends on tumour contrast, size, shape, and heterogeneity [105]. It may be used only as an initial guidance for a subsequent manual delineation. Adaptive thresholding approaches accounting for both contrast and size are more appropriate although they require a scanner specific phantom based calibration procedure [105].
3. Image processing methods — these auto-segmentation approaches allow delineation of uptake semi-automatically without prior calibration and have been developed to either guide or generate the tumour volume [105, 109, 110]. According to ESTRO-ACROP [12], tumour volume should be delineated on the CT image, with guidance of the PET. These methods may help to overcome the low signal-to-noise ratio and the poor spatial resolution of PET images. They may explore the image contrast and the spatial resolution of CT; may create combinations of coregistered PET, CT, or MR data sets; or may use deep learning to analyse a large number of imaging features. Some examples include the following six methods: gradient based method, hybrid method, deformable contour models, model-based methods, statistical image methods, multimodality-based methods, and machine learning methods [111–120].
4. Consensus methods — the combination of several segmentation methods improves segmentation accuracy when compared to a single method. Additionally, it compensates the weaknesses of individual methods and, therefore, may be advantageous for RT planning [121]. Currently, three consensus algorithms are available: majority vote (MJV), simultaneous truth and performance level estimation (STAPLE), and automatic decision tree-based learning algorithm for advanced segmentation (ATLAAS) [122–125].

It has been shown that consensus methods improve accuracy and reproducibility in volume segmentation compared to all separate segmentation methods in different experimental circumstances [121, 126, 127].

The state-of-the-art 2-[^18^F]FDG PET auto-segmentation algorithms, relying on advanced image analysis paradigms, seem to be more accurate than approaches based on manual methods and 2-[^18^F]FDG activity thresholds [105, 106]. However, optimization to scanning conditions, tumour type, and tumour location is still necessary. Currently, there is no approved method and, therefore, all auto-segmentation contours should be critically verified by a physician [105]. An institutionally well organized manual delineation may cover the needs in clinical routine, albeit potentially more time consuming.

**Table 1:** 2-[^18^F]FDG PET metrics and segmentation methods summary

Segmentation methods	
Manual method	• Manual delineation slice by slice
Threshold-based	• SUVmax > 2.5 • >40% SUVmax within the lesion
Advanced image segmentation approaches	• Gradient-based • Hybrid • Deformable contours • Model-based • Statistical • Multimodality-based • Machine learning/deep learning
Consensus algorithms	• Majority vote (MJV) • Simultaneous Truth and Performance Level Estimate (STAPLE) • Automatic decision Tree-based Learning Algorithm for Advanced Segmentation (ATLAAS)

## Documentation/reporting

The interpretation and reporting of 2-[^18^F]FDG PET/CT scans should be done by trained and certified nuclear medicine physician, or a radiologist trained in 2-[^18^F]FDG PET/CT, and with experience in lung malignancies. One combined report including both PET and CT information, or two separate reports for each imaging modality with a summary of the main findings and an integrated conclusion, may be written, according to local circumstances and national reimbursement policies.

The following aspects should be included in a structured report: (1) patient and study identification; (2) clinical information (including the question from the referring clinician, complementary information obtained from the medical history or data collected from the clinical process); (3) procedure including the administered radiopharmaceutical activity, route of administration, uptake time, blood glucose level, PET scanner type, field of view, CT protocol (low dose or dedicated), additional imaging acquisition (e.g. respiratory-gating or delayed thoracic images), details on administered intravenous contrast, ancillary medications, reconstruction technique, and if the PET/CT was performed for RT planning; (4) comparison studies used for correlation; (5) main findings described by order of importance (may follow the TNM staging classification, anatomic site, or hybrid formats); and (6) summary and final impression aiming to answer to the clinical question, to mention the TNM staging in the initial evaluation, to classify the study as complete metabolic response, partial metabolic response, stable disease, pseudo-progression or progressive metabolic disease in restaging, and to provide guidance to the referring doctor.

When using PET/CT scans for delineation, the person performing the delineation should be trained in 2-[^18^F]FDG PET/CT image interpretation. Moreover, it is advisable to document the method of delineation (manual or automatic), including whenever appropriate the threshold used and/or other methodology related parameters (e.g. %SUVmax) to facilitate a second definition of the GTV if necessary (see “27”).

## Equipment specifications, quality control, and radiation safety in imaging

The EANM/SNMMI procedural guidelines for tumour imaging apply for quality control of PET [42, 82]. Also, it is recommended to adhere to the EANM Research Ltd (EARL© http://earl.eanm.org/cms/website.php) accreditation program, which is aimed at harmonizing quantification among different equipment in a wide range of tumour types and is available for 2-[^18^F]FDG PET/CT and PET/MRI [128, 129].

It is recommended that the PET/CT equipment used for RT is in accordance with the requirements for RT planning, including the flat table-top, positioning devices, laser systems, and increased gantry diameter [85]. According to the national and/or international guidelines, the quality control of the PET/CT hardware should also include the quality control of the CT, the PET, and the PET/CT alignment [85, 130, 131]. In the RT context, it is required that the quality control follows the RT recommendations, including table positioning and movement, and laser geometry and accuracy [83, 132].

When put into perspective to the dose received from external beam RT, the radiation dose to patients from PET/CT imaging is negligible. The majority (40–60%) of the radiation exposure of technologists is related to 2-[^18^F]FDG preparation, injection, and patient positioning [133, 134].

Measures to reduce the personnel exposure to radiation should be promoted, and some examples include patient instruction before 2-[^18^F]FDG injection, trained staff in positioning patients, and room preparation prior to patient arrival [84].

For tumour delineation purposes, it is recommended to review the acquired images, namely the alignment of the CT and PET components. Then, the images should be transferred to the RT planning system to enable the final display of the PET/CT images on the planning computer. It is important that each part of the process has undergone appropriate quality assurance testing and that the complete process has been validated.

## Safety, infection control, and patient education concerns

Imaging should follow local safety protocols, but some guidance may be obtained from the “American College of Radiology Position Statement on Quality Control and Improvement, Safety, Infection Control and Patient Education” [135].

## Recent developments considered for future application

### Imaging tumour metabolism and dose painting

Dose painting is a sophisticated approach to selectively deliver dose to different parts of a tumour, including delivery of higher doses to treatment-resistant areas, rather than escalating the dose to the whole tumour [136]. Usually, areas of high pre-treatment 2-[^18^F]FDG uptake within the primary tumour are considered to be more aggressive. Therefore, these areas may be considered the target for dose-escalation [109, 137, 138].

Defining the biologic target volume, i.e. tumour subvolumes requiring a higher or lower dose based on the tumour microenvironment, is a crucial step in RT planning for dose painting and possibly partial dose-escalation, termed boosting. Several methods for pre-treatment segmentation have been proposed, but none of them has been proven superior to another [104, 106, 139]. However, there are some promising results. For instance, the Netherlands randomized phase II PET-boost trial (NCT01024829) [140] showed the feasibility of dose-escalation using an integrated boost to the primary tumour or high 2-[^18^F]FDG uptake regions (>50% SUVmax) whilst keeping the pre-defined dose constraints. In this trial, the dose could be escalated to at least 72 Gy in 75% of patients, without increasing the dose to the OAR.

### Intermediate/mid-treatment 2-[^18^F]FDG PET/CT and adaptive RT

Image-based adaptive RT was initially introduced in an effort to overcome the challenge of tumour motion, but it also enabled an earlier assessment of treatment response [141–143]. It offers an opportunity to identify ineffective therapies and switch to an alternative treatment regimen, preventing futile radiation toxicity. Interim imaging can be performed anytime during the scheduled treatment duration. Several authors have demonstrated that 2-[^18^F]FDG activity changes remarkably during the course of RT. They found that change in mid-treatment 2-[^18^F]FDG activity correlated with post-RT response, which was predictive of overall survival [75, 144–146]. Wang et al. [75] concluded that a 75% decrease of SUV predicted overall survival and 2-year progression free survival (hazard ratio of 0.97 for both). The prospective study RTEP1 analysed 2-[^18^F]FDG PET/CT examinations performed during thoracic RT (the first PET was performed before the first RT fraction, and five additional PET scans were performed after each 14–16 Gy of dose up to the total dose of 70 Gy), given either alone or with chemotherapy [147]. They observed an average 50% decrease in SUVmax at approximately 40–45 Gy (i.e., during week 5 of RT). The subsequent multicenter RTEP2 study (NCT01261598) [148] demonstrated the prognostic value of 2-[^18^F]FDG PET/CT during curative-intent RT with or without concomitant chemotherapy in patients with NSCLC. The SUVmax of the PET2 scan, performed during the fifth week of treatment, was the single variable predictive of death or tumour progression at 1 year in multivariate analysis.

Additionally, the prospective phase 2 RTOG1106 trial (NCT01190527) [149] in patients with locally advanced NSCLC showed the feasibility of dose escalation to persistent 2-[^18^F]FDG avid tumour seen on mid-treatment 2-[^18^F]FDG PET/CT. The interim analysis led to an improved 2-year loco-regional tumour control rate, reaching an infield and overall local regional tumour controls rate of 82% and 62%, respectively. The final results of this trial are expected by the end of 2021. However, a phase 3 study will be required before this adaptive approach starts being used in standard clinical care.

Other clinical trials are ongoing, including the following: NCT02473133, NCT01507428, NCT01261598, NCT01261585, NCT01576796, and NCT02354274.

### Other non-2-[^18^F]FDG radiopharmaceuticals

PET tracers other than 2-[^18^F]FDG have a potential role in imaging tumour biology and heterogeneity, through the evaluation of hypoxia, proliferation, and vascularization. The most commonly used hypoxia tracers are [^18^F]FMISO, [^18^F]HX4 and [^18^F]FAZA. [^18^F]FLT can be used to study proliferation. Since hypoxia is a marker of radioresistance and high proliferation areas that are presumably more aggressive, such tumour areas may require dose escalation [150–156]. Although still under investigation, this complementary information could be used in RT dose-reduction or escalation through dose-painting (see “Imaging tumour metabolism and dose painting”.).

In patients with locally advanced stage III NSCLC, the probability of local control remains low (34% at 1 year without immunotherapy) [157]. A prospective phase II multicentre dose escalation study applying [^18^F]FMISO in NSCLC in hypoxic sub-volumes (RTEP5, NCT 01576796), showed the feasibility of escalating dose up to 86 Gy. The response rate at 3 months was 57% (95% confidence interval [CI], 43%–71%) using RECIST 1.1. Disease-free survival and overall survival at 1 year were 86% (95% CI, 77%–96%) and 63% (95% CI, 49–74%), without significant toxicity. After 3 years of follow-up, the authors found that in [^18^F]FMISO-positive patients, the RT boost increased median overall survival by 11.2 months [158, 159].

### PET/MRI

There are few studies comparing 2-[^18^F]FDG PET/MRI and 2-[^18^F]FDG PET/CT in lung cancer patients, but both seem to show similar high diagnostic performance [160–162]. Nevertheless, MRI of the chest or sub-regions of interest could be added to the workup in cases with chest wall infiltration, superior sulcus tumours (including Pancoast tumours) or para-spinal tumours [163]. To allow a co-registered planning, MRI sequences should be acquired in the RT planning position.

### Radiomics

Radiomics is an emerging field with significant potential for prognostic stratification, RT planning, and response assessment in patients with lung cancer. Radiomics involves the extraction of a large number of quantitative features from medical images using advanced imaging processing and analysis tools, and it is actively explored in lung cancer [164–167]. The integration of artificial intelligence in this radiomic-driven pipeline may also allow mainstreaming their use in clinical practice [168, 169]. Some studies indicate that an analysis of pretreatment 2-[^18^F]FDG PET/CT images based on the use of radiomics may allow to predict local control for patients undergoing SBRT [166, 170]. A recent retrospective multicentre trial study [170] showed that both PET and CT features were predictive of local control, with a predictive model combining two PET features reaching a sensitivity and specificity of 100% and 81%, respectively. Another group found that specific PET features closely correlated to tumour volume definition, specifically, in larger tumours [171]. Due to the variability in acquisition and reconstruction protocols, a “Radiomics Quality Score” was created in order to harmonize the radiomic feature calculation methods and protocols, enabling comparison between different studies [172–174].

## Supplementary information

The Society of Nuclear Medicine and Molecular Imaging (SNMMI) is an international scientific and professional organization founded in 1954 to promote the science, technology, and practical application of nuclear medicine. The European Association of Nuclear Medicine (EANM) is a professional non-profit medical association that facilitates communication worldwide between individuals pursuing clinical and research excellence in nuclear medicine. The EANM was founded in 1985. SNMMI and EANM members include physicians, radiologists, technologists, and scientists specializing in the research and practice of nuclear medicine.

The European Society for Radiotherapy & Oncology (ESTRO) was founded in 1980, and is a non-profit scientific organisation that fosters the role of radiation oncology in order to improve patients’ care in the multimodality treatment of cancer. With over 6,500 members inside and outside Europe, ESTRO supports all the radiation oncology professionals in their daily practice. ESTRO members include radiation oncologists, medical physicists, radiobiologists and radiation technologists and members of the wider oncology community. Its mission is to promote innovation, research, and dissemination of science through congresses, special meetings, educational courses and publications.

The SNMMI and EANM periodically define new guidelines for nuclear medicine practice to help advance the science of nuclear medicine and improve the quality of service to patients throughout the world. Existing practice guidelines are reviewed for revision or renewal, as appropriate, on their fifth anniversary or sooner, if indicated. Each practice guideline, representing a joint policy statement by the SNMMI/EANM, has undergone a thorough consensus process in which existing evidence has been subjected to extensive review. The SNMMI and EANM recognize that the safe and effective use of diagnostic nuclear medicine imaging requires specific training, skills, and techniques, as described in each document. Reproduction or modification of the published practice guideline by those entities not providing these services is not authorized.

These guidelines represent an educational tool designed to assist practitioners in providing appropriate care for patients. They are not inflexible rules or requirements of practice and are not intended, nor should they be used, to establish a legal standard of care. For these reasons, and those set forth below, both the SNMMI and the EANM caution against the use of these guidelines in litigation in which the clinical decisions of a practitioner may be called into question.

The ultimate judgment regarding the propriety of any specific procedure or course of action must be made by the physician or medical physicist in light of all the circumstances presented. Thus, there is no implication that an approach differing from the guidelines, standing alone, is below the standard of care. To the contrary, a conscientious practitioner may responsibly adopt a course of action different from that set forth in the guidelines when, in the reasonable judgment of the practitioner, such course of action is indicated by the condition of the patient, limitations of available resources, advances in knowledge or technology subsequent to publication of the guidelines, local regulatory requirement, or reimbursement frameworks. The practice of medicine includes both the art and the science of the prevention, diagnosis, alleviation, and treatment of disease. The variety and complexity of human conditions make it impossible to always reach the most appropriate diagnosis or to predict with certainty a particular response to treatment.

Therefore, it should be recognized that adherence to these guidelines will not ensure an accurate diagnosis or a successful outcome. All that should be expected is that the practitioner will follow a reasonable course of action based on current knowledge, available resources, and the needs of the patient to deliver effective and safe medical care. The sole purpose of these guidelines is to assist practitioners in achieving this objective.

Members of the EANM Oncology Committee (Sofia C. Vaz, Judit A. Adam, Ken Herrmann, Lioe-Fee de Geus-Oei), EANM Physics Committee (Dimitris Visvikis), EANM Technologists Committee (Andrea Santos), the SNMMI representative (Heiko Schöder) and the Advisory Committee on Radiation Oncology Practice (ACROP) of ESTRO (Bernard Dubray, Wouter van Elmpt, Yolande Lievens, Esther G.C. Troost), invited experts from Europe (Roberto C. Delgado Bolton, Pierre Vera) and Australia (Rodney J. Hicks) took part in developing this guideline.

## Abbreviations

2-[^18^F]FDG: 2-[^18^F]fluoro-2-deoxy-D-glucose
4-D: Four-dimensional
ACROP: Advisory Committee in Radiation Oncology Practice
AJCC: American Joint Commission On Cancer
CRT: Chemoradiotherapy
CTV: Clinical target volume
EANM: European Association of Nuclear Medicine
EBRT: External beam radiation therapy
EBUS: Endobronchial ultrasound
EUS: Endoscopic ultrasound
EORTC: European Society for Radiotherapy and Oncology
ESMO: European Society of Medical Oncology
ESTRO: European Organisation for Research and Treatment Of Cancer
GTV: Gross tumour volume
IAEA: International Atomic Energy Agency
IMRT: Intensity modulated radiation therapy
MRI: Magnetic resonance imaging
MTV: Metabolic tumour volume
NCCN: National Comprehensive Cancer Network
NSCLC: Non-small cell lung cancer
OAR: Organs at risk
PET/CT: Positron emission tomography / computed tomography
PTV: Planning target volume
RT: Radiotherapy
SCLC: Small cell lung cancer
SBRT/SART: Stereotactic body radiation therapy or stereotactic ablative radiation therapy
SNMMI: Society of Nuclear Medicine and Molecular Imaging
SUVmax: Maximum standardized uptake value
VMAT: Volumetric modulated arc therapy

## Declarations

### Disclaimer

This guideline summarizes the views of the EANM Oncology and Theranostics Committee, the SNMMI and ESTRO. It reflects recommendations for which the EANM/SNMMI/ESTRO cannot be held responsible. The recommendations should be taken into context of good practice of nuclear medicine and radiation oncology and do not substitute for national and international legal or regulatory provisions.
