# Correction to: Joint EANM/SNMMI/ESTRO practice recommendations for the use of 2-[^18^F]FDG PET/CT external beam radiation treatment planning in lung cancer V1.0

**DOI:** 10.1007/s00259-022-05810-z

**Published:** 2022-04-28

**Authors:** Sofia C. Vaz, Judit A. Adam, Roberto C. Delgado Bolton, Pierre Vera, Wouter van Elmpt, Ken Herrmann, Rodney J. Hicks, Yolande Lievens, Andrea Santos, Heiko Schöder, Bernard Dubray, Dimitris Visvikis, Esther G. C. Troost, Lioe-Fee de Geus-Oei

**Affiliations:** 1 Nuclear Medicine Radiopharmacology, Champalimaud Centre for the Unkown, Champalimaud Foundation, Lisbon, Portugal; 2 Department of Radiology, Leiden University Medical Center, Leiden, The Netherlands; 3 Department of Radiology and Nuclear Medicine, Amsterdam University Medical Center, Amsterdam, The Netherlands; 4 Department of Diagnostic Imaging (Radiology) and Nuclear Medicine, University Hospital San Pedro and Centre for Biomedical Research of La Rioja (CIBIR), Logroño (La Rioja), Spain; 5 Henri Becquerel Cancer Center, QuantIF-LITIS EA 4108, Université de Rouen, Rouen, France; 6 Department of Radiation Oncology (MAASTRO), GROW – School for Oncology, Maastricht University Medical Centre, Maastricht, The Netherlands; 7 Department of Nuclear Medicine, University of Duisburg-Essen and German Cancer Consortium (DKTK) University Hospital Essen, Essen, Germany; 8 The Sir Peter MacCallum Department of Oncology, University of Melbourne, Melbourne, Australia; 9 Radiation Oncology Department, Ghent University Hospital and Ghent University, Ghent, Belgium; 10 Nuclear Medicine Department, CUF Descobertas Hospital, Lisbon, Portugal; 11 Molecular Imaging and Therapy Service, Memorial Sloan Kettering Cancer Center, New York, USA; 12 Department of Radiotherapy and Medical Physics, Centre Henri Becquerel, Rouen, France; 13 QuantIF-LITIS EA4108, University of Rouen, Rouen, France; 14 LaTIM, INSERM, UMR1101, Brest, France; 15 Department of Radiotherapy and Radiation Oncology, Faculty of Medicine and University Hospital Carl Gustav Carus, Technische Universität Dresden, Dresden, Germany; 16 OncoRay – National Center for Radiation Research in Oncology, Faculty of Medicine and University Hospital Carl Gustav Carus, Technische Universität Dresden, Helmholtz-Zentrum Dresden-Rossendorf, Dresden, Germany; 17 Helmholtz-Zentrum Dresden - Rossendorf, Institute of Radiooncology - OncoRay, Dresden, Germany; 18 National Center for Tumor Diseases (NCT), Partner Site Dresden, Germany: German Cancer Research Center (DKFZ), Heidelberg, Germany; Faculty of Medicine and University Hospital Carl Gustav Carus, Technische Universität Dresden, Dresden, Germany; Helmholtz Association / Helmholtz-Zentrum Dresden – Rossendorf (HZDR), Dresden, Germany; 19 German Cancer Consortium (DKTK), Partner Site Dresden, and German Cancer Research Center (DKFZ), Heidelberg, Germany

**Correction to: European Journal of Nuclear Medicine and Molecular Imaging (2022) 49:1386–1406**

**https://doi.org/10.1007/s00259-021-05624-5**

The authors regret that there is an error in their original article. They found a small mistake in the abbreviations, because the meaning of ESTRO and EORTC has been interchanged. It says:

-EORTC = European Society for Radiotherapy and Oncology

-ESTRO = European Organisation for Research and Treatment of Cancer

But it should be:

-ESTRO = European Society for Radiotherapy and Oncology

-EORTC = European Organisation for Research and Treatment of Cancer

